# Kearsing’s Foil

**Published:** 1883-11

**Authors:** 


### KEARSING'S FOIL.

For some years I have been using the condensed gold foil manufactured by E. Kearsing, 101 Hoyt Street, Brooklyn, and always with satisfaction. It is very cohesive when annealed, without any of the stiffness and unadaptability so common in ordinary cohesive gold. I can most unhesitatingly recommend it.

C. F. W. B.

